# Correction: Liquid electrolyte chemistries for solid electrolyte interphase construction on silicon and lithium-metal anodes

**DOI:** 10.1039/d3sc90171h

**Published:** 2023-09-14

**Authors:** Sewon Park, Saehun Kim, Jeong-A Lee, Makoto Ue, Nam-Soon Choi

**Affiliations:** a Department of Chemical and Biomolecular Engineering, Korea Advanced Institute of Science and Technology (KAIST) 291 Daehak-ro, Yuseong-gu Daejeon 34141 Republic of Korea nschoi@kaist.ac.kr; b Research Organization for Nano & Life Innovation, Waseda University 513 Waseda-tsurumaki-cho, Shinjuku-ku Tokyo 162-0041 Japan

## Abstract

Correction for ‘Liquid electrolyte chemistries for solid electrolyte interphase construction on silicon and lithium-metal anodes’ by Sewon Park *et al.*, *Chem. Sci.*, 2023, https://doi.org/10.1039/d3sc03514j.

The authors regret that there is an error in Section 3.1.2 of the original article. The correct sentence is shown below.

Multilayer SEI structuring is further discussed in Section 3.3.3.

The Royal Society of Chemistry apologises for these errors and any consequent inconvenience to authors and readers.

## Supplementary Material

